# Comparative Analysis of Restorative Interior Design Elements: Screen-Based Versus Virtual Reality Evaluations for Future Medical Treatment Prospects

**DOI:** 10.3390/ijerph22010044

**Published:** 2024-12-31

**Authors:** Alp Tural, Elif Tural

**Affiliations:** School of Design, Virginia Tech, Blacksburg, VA 24061, USA; etural@vt.edu

**Keywords:** restorative interior environments, virtual reality, mental health treatment, biophilic design

## Abstract

Given the increasing prevalence of anxiety and depression, this research aims to identify design features that enhance the sense of restoration, with the goal of supporting mental and behavioral healthcare facility design. This study employed both screen-based and virtual reality (VR) stimuli to evaluate the perceived restorativeness of different interior settings. The key variables analyzed included window view access, view content, materiality, and room geometry. Thirty-five undergraduate and graduate students assessed 16 distinct interior environments. Findings indicate that the VR presentations generally produced higher restorativeness scores compared with screen-based presentations, though this effect varied across stimuli. Repeated-measures ANOVA revealed that larger windows consistently correlated with higher restorativeness scores in both presentation modes. Views of water were rated as most restorative, followed by wooded areas. Natural materials were perceived as significantly more restorative than other materials, particularly in VR presentations. Varied ceiling designs, especially vaulted ceilings, were associated with evaluations of higher restorativeness compared with flat ceiling designs, with this effect more pronounced in VR. This research underscores the potential of VR technology to simulate and assess interior design interventions, offering insights into creating more effective and personalized restorative environments in mental health treatment facilities. The findings can inform evidence-based design strategies for healthcare spaces, supporting treatment processes and patient well-being.

## 1. Introduction

The design of interior spaces, particularly in healthcare settings, has garnered increasing attention due to its potential impact on human well-being and restoration. This study investigates the influence of specific interior design elements on restorative quality, considering patient mental health and well-being at mental health treatment facilities. This research is especially timely given the substantial increase in the prevalence of anxiety and depression due to recent global events, underscoring the need for carefully designed restorative environments [1,2,3].

Unlike ‘client-focused’ approaches to interior design in long-term healthcare settings [4], for short-term treatment, medical professionals in mental health treatment, healing, and psychiatry centers traditionally utilize their offices or examination rooms as the primary spaces for attending to patients who do not require a higher level of care, such as those seeking ambulatory anxiety treatment. These spaces, while functional, often serve as multi-purpose areas where clinicians conduct assessments, consultations, and therapy sessions in a clinical setting. Despite their utility and technological advancements, these traditional spaces may lack the specialized features and restorative atmosphere necessary to fully support the emotional and psychological needs of patients undergoing treatment. Additionally, the inherent clinical nature of these environments may inadvertently contribute to feelings of anxiety or discomfort in some patients, underscoring the importance of designing more tailored and conducive spaces that address the unique challenges and complexities of mental health interventions.

Furthermore, from the building design and construction perspective, it is crucial to acknowledge that creating restorative interior environments that fully meet the diverse treatment and emotional needs of the majority of patients is a significant challenge, particularly in older buildings. Constraints include limited access to natural light, restricted outdoor views, issues with view content and quality, difficulties in retrofitting controllable light sources, and outdated finishes. These challenges highlight the need for innovative approaches to interior design in healthcare settings.

## 2. Virtual Reality as an Innovative Approach

Virtual reality (VR) presents significant potential to overcome these challenges by offering immersive 3D interior settings where spatial aspects of the environment can be manipulated and tailored to the needs of each patient, caregiver, and service provider [5], for treatment purposes. VR technology and tools can facilitate the creation of customizable virtual environments that replicate and substitute real-world scenarios, providing individuals with a sense of presence and immersion. Especially for those who do not have immediate access to such environments, VR can offer a practical solution [6]. These virtual settings can be adjusted to eliminate or gradually mitigate environmental triggering stimuli.

The use of VR as a research platform for investigating human behavior and environmental preferences is well established, with studies exploring user experiences, spatial perceptions, and physiological responses across various design disciplines [7,8,9,10,11,12,13,14]. In restorative and biophilic design research, VR has been used both as a research tool to study the different types of stimuli and as a method to collect data in situations where direct access to natural environments or research settings is not possible [15].

In psychiatric care, VR-based exposure therapy (VRET) has gained traction for treating phobias and social anxiety [16,17,18,19,20]. However, comprehensive studies are needed to evaluate the effectiveness of immersive VR interior environments compared with traditional methods in existing treatment spaces.

## 3. Theoretical Background on Sense of Restoration

The notion of fostering restoration through interior design draws upon insights from various theoretical frameworks across environmental psychology, evolutionary biology, and cultural studies. Key theories underpinning this research include the following:

Biophilic design theory, rooted in the biophilia hypothesis [21], suggests an innate human affinity towards nature and natural processes. The incorporation of natural elements in interior design is hypothesized to stimulate physiological and psychological responses that promote well-being and restoration [22].

Ulrich’s stress recovery theory (SRT) underscores measurable outcomes and evidence-based design and suggests that natural settings elicit positive emotional responses, hold attention modestly, and inhibit negative thoughts, aiding in stress recovery. Ulrich’s findings have significantly impacted environmental design, particularly in healthcare settings, where the integration of natural elements has been shown to contribute to faster recovery rates and improved patient well-being [23,24,25,26].

Attention restoration theory (ART) also explores the restorative effects of exposure to natural environments but focuses more on the cognitive system and mental fatigue. ART identifies four key components of restorative environments: being away, extent, fascination, and compatibility. These components contribute to mental restoration by providing a sense of escape and engaging the mind in a non-taxing manner [27,28]. Additional perspectives include the Kaplans’ studies on environmental preference, suggesting innate human preferences for environments characterized by coherence, complexity, legibility, and mystery [29]. Prospect–refuge theory draws upon evolutionary psychology to explain preferences for environments with balanced visual access and shelter, while place-attachment theory explores emotional and cognitive bonds that individuals form with places [30].

From a probabilistic perspective, interior designs that strategically leverage these principles are hypothesized to have the potential to create spaces that evoke feelings of comfort, security, and ultimately, restoration [31].

## 4. Literature, Research Gaps, and Objectives

Despite the rich theoretical foundation, meta-analytic studies on the built environment, emotional response, and preference highlight the difficulty of proposing generalizable findings, due to several factors including the subjectivity of human consciousness and varying definitions of emotional constructs [32,33], methodological inconsistencies and contextual differences among studies [29,34,35], oversight of mediating variables such as cognitive functions and performance [36,37], and differences in sensory connection to tested or simulated environments [38].

Previous research on the use of VR in psychiatric care underlined similar limitations due to lack of comparison groups, as well as varying measurements and protocols, hindering the generalization of findings [39,40,41,42].

In light of these challenges, several directions for future research have been suggested in the literature. These include exploring the restorative effect of interiors on younger adults [43], investigating the impact of finish materials on the sense of restoration [44], incorporating objective measures to complement self-reported perceptions [45], replicating findings in diverse contexts, and identifying specific architectural components that influence restorative interior settings [46].

This study aims to address several of these research gaps by investigating the influence of four key design variables on the sense of restoration, utilizing both screen-based stimuli and immersive VR environments. The variables were identified through the restorative design theoretical framework, focusing on the elements contributing to restorative environments. Two of these variables were related to window design, considering the view quality and degree of access to the view. Windows are acknowledged as a form of passive engagement with outdoors and nature [26], making them a crucial element in restorative interior design. The third variable examined the impact of interior surface finish and materials, while the fourth examined the effect of spatial form, specifically focusing on various ceiling designs.

The study objectives were as follows:

1. To investigate the influence of specific interior design elements on restorative quality in a hypothetical healthcare setting, with a focus on young adult populations;
2. To utilize a novel approach combining screen-based data collection and VR environments to examine the relationship between interior design variables and perceived restoration;
3. To explore the impact of view access, view content, materiality, and room geometry on restorative experiences.

This study demonstrates how space can easily be manipulated within a virtual environment to create various settings that can meet different treatment needs. By altering spatial elements such as room size, layout, and visual aesthetics, VR can simulate a range of environments that may enhance patient comfort and treatment outcomes. This flexibility allows the customization of treatment spaces to better align with individual patient requirements, potentially leading to more effective and personalized psychiatric care.

## 5. Research Variables and Hypotheses

This research employed a survey to quantify participants’ perceptions of restoration while they observed interior settings via a computer screen or in VR using a headset. By comparing these viewing methods, this study investigated whether VR’s immersive presentation format yielded higher restoration ratings than screen-based visualizations.

VR simulations can be leveraged for in-person treatments, creating immersive and adaptable treatment spaces that respond more effectively to patients’ needs. Additionally, the findings can provide valuable insights for the use of screen-based visualizations in telemedicine, where remote consultations can benefit from restorative environments.

**H1.**  *Environmental stimuli presented through immersive virtual reality will generate significantly higher restoration ratings compared with screen-based viewing conditions.*

### 5.1. View Access

View access represents the quantifiable portion of window view observable from an occupant’s spatial position. It has emerged as a critical consideration across various built environments, with particular relevance to mental health and well-being. Research suggests that view access may contribute to reduced stress and improved mood in workplace settings, enhanced cognitive function and emotional regulation in educational environments, and potentially accelerated recovery processes in healthcare facilities [24,47,48,49,50]. Research on view access has prompted the development of view access metrics and daylight access standards, such as EN 17037 [51] and LEED quality views [52]; however, there is not yet an established consensus on how those standards should operationalize the metric [53,54]. In this research, conditions of access to the view systematically varied from a solid wall (0%) to three incremental window sizes (33%, 66%, and 100%), to investigate how the degree of the view from a fixed observation point influenced the restorative effects.

**H2.**  *Large window areas will yield significantly higher restoration ratings compared with spaces with limited window area or windowless conditions.*

### 5.2. View Content and View Quality

The literature on restorative environments suggests that the presence of water features, vegetation, and views with moderate complexity that foster curiosity are significant factors affecting well-being. White and colleagues reported that aquatic environments were rated more positively for restoration than green space alone [55]. In indoor environments, the view area, number of layers, and fragmentation of the view have been identified as quantitative measures of view quality [53,54,56]. This research evaluated the relationship between sense of restoration and four categories of natural view content: desert, water, woods, and lawn.

**H3.**  *Particular natural features (e.g., water elements, diverse vegetation) will demonstrate a stronger positive correlation with perceived sense of restoration compared with other natural elements.*

### 5.3. Materiality

Materials can introduce a restorative influence in interior design through various means. From a biophilic perspective, materials can create sensory richness, abstract compositions, or motifs of nature, and they can affect one’s connection to space [57]. Natural materials and geometries (the next variable) in interiors are acknowledged as indirect experiences of nature [44]. Aligning with those principles, this study examined the effects of textured vs. smooth and biophilic vs. conventional materials (brick, concrete, natural, and resilient), as former studies have mainly focused on applications of single materials, surface treatments such as green walls [25,58], and vegetation density.

**H4.**  *Interior environments incorporating biophilic features and sensory-rich materials (including natural materials, textures, and patterns) will generate significantly higher restoration ratings compared with alternative material schemes.*

### 5.4. Room Geometry

Spatial geometric relationships have long been investigated in environmental preference studies [59], with recent neuroarchitecture research offering new insights [60,61,62]. This study explored how interior geometric variations, implemented through ceiling design modifications (curved, flat, vaulted and angled ceiling designs), influences the sense of restoration.

**H5.**  *Non-flat ceiling designs (vaulted, curved, or angled configurations) will generate significantly higher perceived restoration levels than flat ceiling conditions.*

## 6. Materials and Methods

### 6.1. Participants

Thirty-five participants enrolled in the study. The sample comprised college undergraduate and graduate students recruited via the Interior Design program and the Design School email listservs. There was an imbalance in participants’ sex (92% female), reflecting the typical distribution within the program cohort from which most participants were drawn.

The study was approved by the University IRB (IRB 22-322). The study consent document was made available with the digital study signup sheet, and each participant received a $20 gift card at the end of the data collection session, which lasted thirty minutes on average. The participants provided verbal consent declaring their voluntary participation, and they were informed about the data collection process and tools (VR headset and AR glasses) before starting the data collection sessions. Participants were allowed to withdraw their consent from the study at any time.

### 6.2. Study Setting and Procedure

The experiment was conducted in an academic office environment. Window coverings remained closed throughout data collection to ensure optimal VR sensor performance and maintain consistent lighting conditions. Upon arrival, participants were guided through the study procedures and received detailed instructions for both phases.

A single-item self-report measure utilizing a 7-point Likert scale was implemented to evaluate perceived restoration across screen and VR presentations of the settings. This measurement strategy was adopted due to its practical advantages for repeated measures in VR and its validated use in analogous studies [13]. The item ‘Please rate the degree of restorativeness this setting offers you’ was developed to capture the overall self-reported restorative quality, as conceptualized in healthcare design research [63] and environmental preference studies.

Research shows that while multi-item scales provide comprehensive measurement in healthcare facility evaluations [64], single-item self-report measures achieve comparable predictive validity in architectural and design studies [61,65]. This measurement approach adheres to the conventions of environmental psychology while capturing subjective restoration experiences [66]. Also, in this study, survey length was a primary concern, making single-item measures particularly appropriate.

This research employed a within-subjects design where all participants experienced both screen-based and VR conditions. The study variables were presented sequentially rather than in randomized order. This sequential presentation was deliberately chosen to maintain logical coherence in the progression of environmental changes, particularly for variables like view access where incremental changes were important for participant evaluation.

### 6.3. Experimental Stimuli

**Figure 1:**
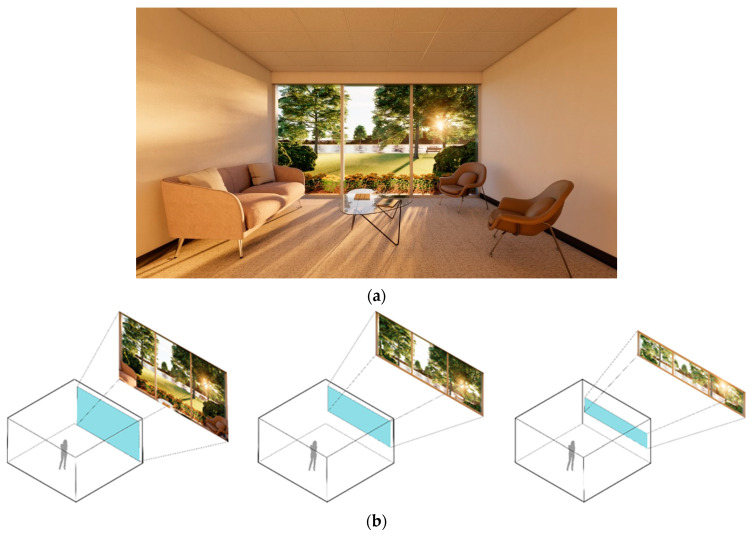
Set 1, view access and 3D-rendered view content: (**a**) Interior rendered view; (**b**) opening proportions: 100%, 66%, and 33%.

**Figure 2:**
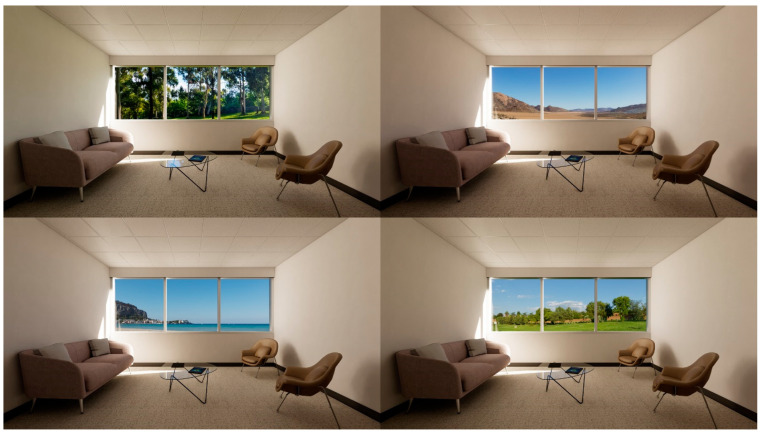
Set 2, view content: 360-degree HDR natural images.

**Figure 3:**
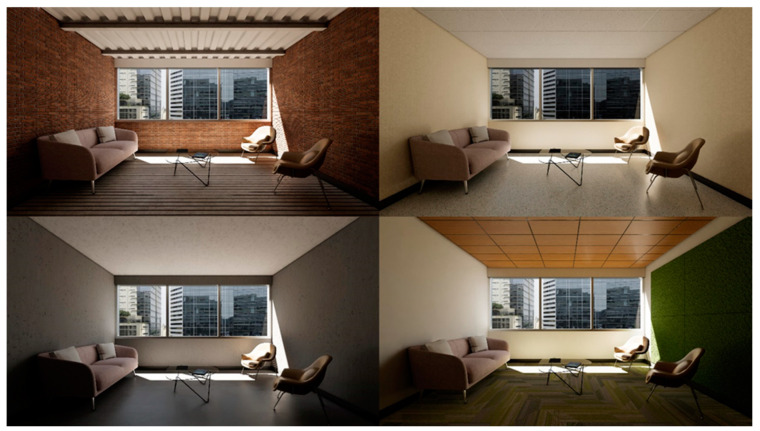
Set 3, materiality.

**Figure 4:**
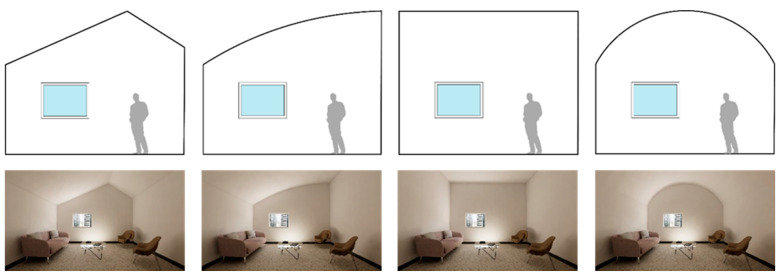
Set 4, room geometry: ceiling form.

Sixteen different interior environments were digitally constructed using Autodesk Revit and rendered via Enscape3D visualization software (Enscape v3.5, Chaos, Sofia, Bulgaria) to achieve photorealistic quality. For screen-based viewing, single-point perspective renderings were generated at 2160 × 1440 pixel resolution (Figure 1, Figure 2, Figure 3 and Figure 4). The camera was placed centrally against the back wall; its position replicated the seated eye height of a 50th-percentile US female. A 100-degree horizontal view angle was implemented to maximize spatial coverage while avoiding distortion. This configuration resulted in balanced surface visibility: lateral walls each occupied 20.5% of total pixels, floor 21%, front wall 20%, and ceiling 17.5%. The slight reduction in ceiling visibility resulted from a five-degree downward camera tilt, simulating natural seated viewing behavior. VR presentations utilized 8192 × 8192 pixel panoramic renders, generated through Enscape’s panoramic export feature. While maintaining the same eye height, the viewing position was offset four feet from the back wall to accommodate 360-degree participant viewing. All spaces had a square floor area of 15 feet by 15 feet and were furnished as a consultation office with consistent elements: a couch, two chairs, and a coffee table. The first three experimental sets used a standard 9-foot ceiling height. The fourth set, examining ceiling geometry variations, extended to 12 feet at its peak while preserving overall room volume. Environmental variables including screen luminance, surface colors, and lighting spectra were standardized across all conditions.

The study environments were divided into four categories, as follows:

Set 1, View Access: The first sequence examined incremental changes in access to outdoor views, ranging from a fully enclosed space to one with complete floor-to-ceiling transparency on the wall perpendicular to the observer (Figure 1). Two intermediate conditions provided 33% and 66% wall apertures, respectively. The outdoor scene featured a natural setting including plants, trees, a water body, and realistically rendered daylight conditions. The arrangement of trees formed a natural boundary while inviting visual discovery. Consistent solar angles were maintained within each set to enhance sensory detail and temporal awareness;

Set 2, View Content: The second sequence included different exterior scenes, while maintaining consistent opening dimensions, interior finishes, and ceiling geometry. Drawing from research on biophilic design and restoration, four distinct environments were presented: a dense woodland offering a permeable boundary, an expansive lawn providing connectedness and spatial extent, a waterscape with distant shoreline facilitating mental escape, and a desert landscape characterized by varied terrain and clear pathways (Figure 2). To maximize visual realism, the exterior scenes were constructed using high dynamic range (HDR) 360-degree photographs. These panoramic images were integrated with the digital interior models, enabling exploration of the views from multiple angles during the VR sessions. Solar angles in the HDR imagery were aligned with the interior lighting conditions to ensure uniform illumination across all variations in the set;

Set 3, Materiality: Material finishes for walls, floors, and ceiling varied while view content and opening dimensions remained unchanged (Figure 3). The window size was maintained at 66% of the front wall area, framing an urban downtown view. The sequence included (a) an adaptive reuse setting of brick, hardwood, and steel decking; (b) a composition with a concrete finish; (c) a biophilic design incorporating green wall panels, timber acoustic ceiling, and nature-patterned carpeting; and (d) a setting featuring textured wall surfaces and resilient flooring. The sizes of the green wall and biophilic intervention areas were controlled, as findings have suggested adverse effects of larger-sized biophilic interventions [67].

Set 4, Room Geometry: To emphasize geometric variations, this series featured a reduced, asymmetrically positioned window revealing the urban vista used in the previous set. Interior surfaces received uniform treatment with matte white paint on gypsum board, except for the flooring which matched the earlier sets—a design decision supported by research indicating that floor characteristics have less impact on spatial perception than wall and ceiling treatments [68]. Maintaining a consistent maximum height of 10 feet, the ceiling configurations explored four distinct geometries: flat, vaulted, curved, and angled forms.

### 6.4. Data Collection Tools

For the VR session in this study, two viewing options were provided: a Meta Quest Pro wireless VR headset or Xreal Air 2 AR glasses paired with an iPad. This dual-platform approach was implemented to accommodate neurodiverse users and those with sensory sensitivities, addressing known VR-related challenges such as reality disconnection anxiety, sensory overwhelm, and physical discomfort [69,70]. The VR headset included detachable side-light shields for customizable immersion levels. While all participants were instructed about shield removal, they unanimously chose to maintain them. The viewing experience differed between platforms: VR users could explore environments through natural head movements, while AR users viewed a 130-inch virtual screen, navigating the 360-degree views through iPad rotation or touch interaction. To ensure optimal performance, the VR headset was connected wirelessly to a PC via a dedicated router, running visualizations through Steam Media Player’s panoramic viewer. The AR glasses maintained a direct cable connection to an iPad Pro, accessing panoramic content through pre-loaded browser tabs.

#### 6.4.1. Screen Stimuli Session

During the screen-based session, viewing distance was maintained at 25 inches, with participants able to adjust seat height to their preference. The session included eighteen slides, beginning with two 25 s informational slides outlining the study protocols and survey question. The subsequent stimulus slides were presented for 18 s each, with exposure durations determined by pilot study findings (*n* = 6). In the screen-based presentations, a fixed-viewpoint approach was implemented to support eye-tracking analyses, which will be reported in a separate paper focusing on attention patterns and visual behavior in restorative environments.

#### 6.4.2. VR/AR Session

After completing the screen-based session, participants selected either the VR headset or AR glasses for the immersive visualization phase. The chosen device allowed unrestricted viewing and rotational movement. For VR users, lens spacing was adjusted to match individual interpupillary distances. Unlike the timed screen-based session, participants controlled their viewing duration for each environment, indicating readiness to advance at their own pace. Among the 35 participants, 1 opted to use the AR glasses rather than the VR headset.

## 7. Results

### 7.1. Comparison of Display Modalities: Screen and Virtual Reality

H1: Environmental stimuli presented through immersive virtual reality will generate significantly higher restoration ratings compared with screen-based viewing conditions. The relationship between display modality and perceived restoration is summarized in Table 1, which presents mean ratings and cross-modal correlations for four distinct environmental categories: View Access, View Content, Materiality, and Room Geometry. Results indicated that VR presentations led to increased restoration scores compared with screen-based viewing, across all stimulus types. Notably, the “100% View Access” condition yielded the highest restorativeness ratings in both the screen-based (M = 6.37, SD = 0.690) and VR (M = 6.51, SD = 0.818) presentations. Additionally, strong positive correlations were observed between screen-based and VR ratings for most stimuli, with particularly high correlations in the Materiality set (e.g., r = 0.811, *p* < 0.01 for the natural finishes).

A series of paired-samples *t*-tests were conducted to compare perceived restorativeness scores between screen-based and VR presentations of environmental stimuli. The results partially supported the hypothesis that perceived restoration would be significantly higher in VR compared with screen-based presentations.

Significant differences were found for several environments, with VR presentations yielding higher restorativeness scores. These included the 33% façade opening (t(34) = −1.455, *p* = 0.155, d = −0.246), the desert view (t(34) = −2.741, *p* = 0.010, d = −0.463), the water view (t(34) = −1.712, *p* = 0.096, d = −0.289), the brick finishes (t(34) = −3.011, *p* = 0.005, d = −0.509), and the natural finishes (t(34) = −2.528, *p* = 0.016, d = −0.427). However, for some environments, the screen-based presentations were associated with higher restorativeness scores. These included the room with no façade opening (t(34) = 2.518, *p* = 0.017, d = 0.426) and the room with the lawn view (t(34) = 1.178, *p* = 0.247, d = 0.199), although the latter was not statistically significant.

Several environments showed no significant differences between the on-screen and VR presentations, including the 66% façade opening (t(34) = −0.780, *p* = 0.441, d = −0.132), 100% façade opening (t(34) = −1.152, *p* = 0.257, d = −0.195), the wooded area view (t(34) = 0.172, *p* = 0.865, d = 0.029), concrete finishes (t(34) = −0.552, *p* = 0.585, d = −0.093), and various room geometries (curved: t(34) = −0.361, *p* = 0.721, d = −0.061; angled: t(34) = 0.000, *p* = 1.000, d = 0.000; vaulted: t(34) = −1.872, *p* = 0.070, d = −0.316).

Effect sizes ranged from small to medium, with the largest effect observed for the room with brick finishes (d = −0.509), favoring VR presentation.

These results reveal that while VR presentations tended to produce higher perceived restorativeness for some environments, the effect was not consistent across all stimuli. The hypothesis is partially supported, with the relationship between presentation mode and perceived restoration appearing to be dependent on the specific environmental context.

### 7.2. Effects of View Access, View Content, Materiality and Room Geometry on Perceived Sense of Restorativeness

One-way repeated-measures analyses of variance were conducted using SPSS (Version 29) to examine the effects of view access, view content, materiality, and room geometry on perceived restoration. Given the mixed findings for H1, screen-based and VR conditions were analyzed separately. Each environmental variable included four levels. The assumption of normality was verified using normal Q-Q plots. Table 2 and Table 3 present the RM ANOVA results for both display modalities, demonstrating substantial effect sizes across all models.

**Figure 5:**
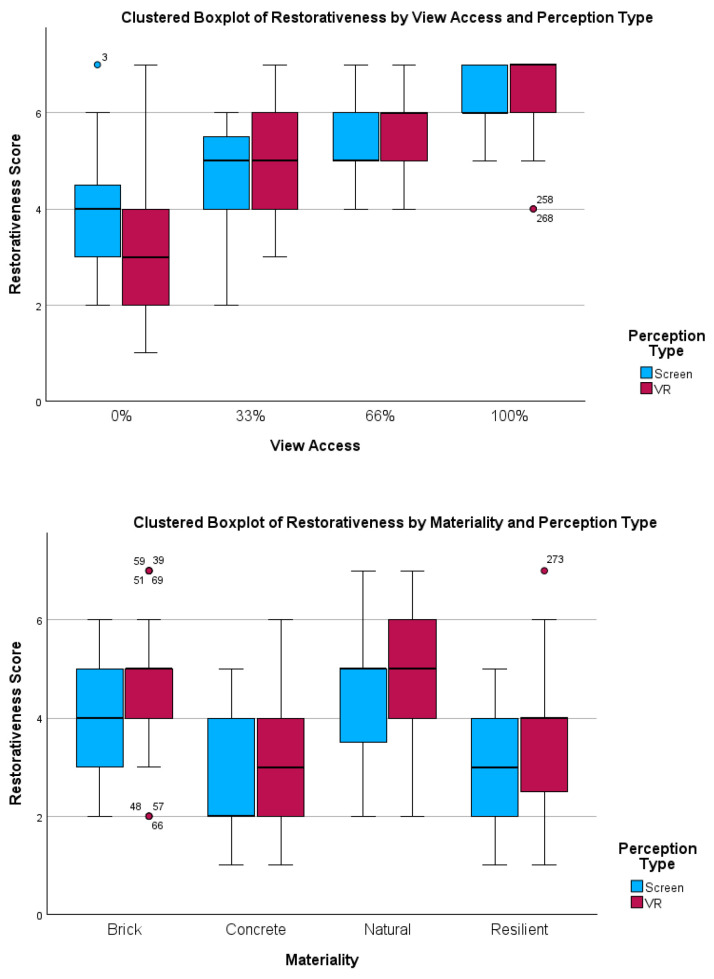

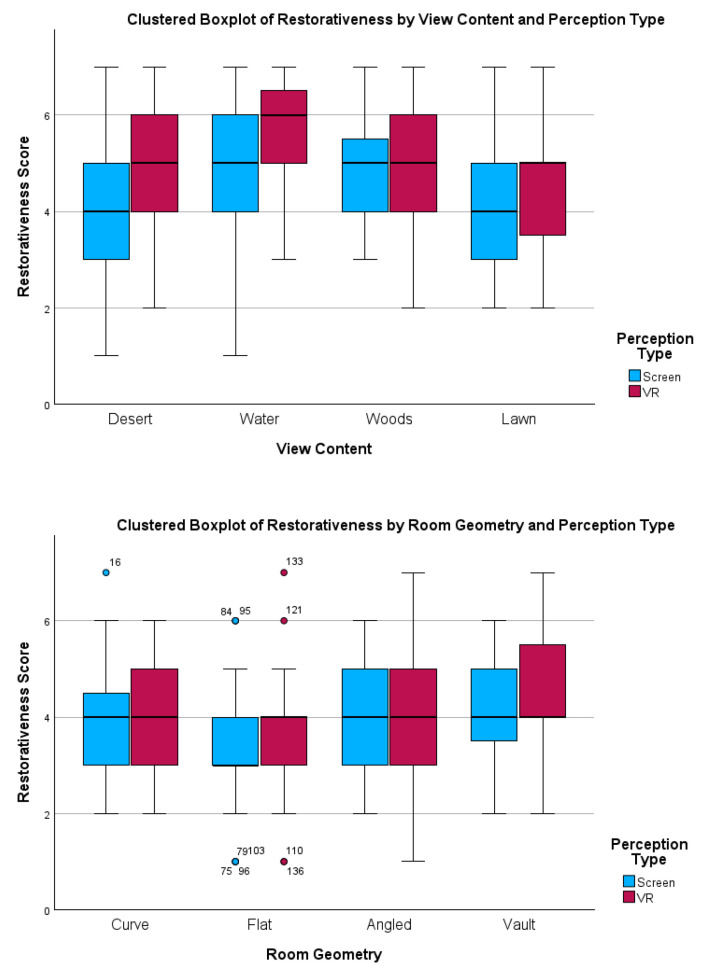
Clustered boxplots of restorativeness scores by variable and perception type.

Table 4 provides the results from the pairwise comparisons of these variables, for both screen- and VR-based evaluations. Figure 5 shows four clustered boxplots representing restorativeness scores across two perception types.

#### 7.2.1. View Access

H2_screen: Large window areas will yield significantly higher restoration ratings, compared with spaces with limited window area or windowless conditions.

One-way repeated-measures ANOVA was conducted to examine the effect of view access/window size on perceived restorativeness scores. Mauchly’s test indicated that the assumption of sphericity was violated, χ^2^(5) = 20.965, *p* < 0.001; therefore, degrees of freedom were corrected using Greenhouse–Geisser estimates of sphericity (ε = 0.698).

The results showed a significant main effect of window size on perceived restorativeness, F(2.095, 71.234) = 59.374, *p* < 0.001, partial η^2^ = 0.636, indicating a large effect size. These findings align with the hypothesis that increased window areas correlates with higher perceived restoration ratings, compared with limited window area or windowless conditions.

Post hoc tests using the Bonferroni correction revealed that all pairwise comparisons between the four levels of window size (0%, 33%, 66%, and 100%) were statistically significant (*p* < 0.05). Perceived restorativeness scores increased significantly with each increase in window size. The largest mean difference was observed between the 0% and 100% window conditions (MD = 2.486, SE = 0.202, *p* < 0.001, 95% CI [1.919, 3.053]).

The results provided robust support for the hypothesis, revealing a strong positive relationship between window size and perceived restoration. The analysis showed a clear linear progression; the restoration ratings systematically increased with expanding window dimensions, from windowless conditions to full-height glazing.

H2_VR: Large window areas will yield significantly higher restoration ratings, compared with spaces with limited window area or windowless conditions.

Mauchly’s test indicated that the assumption of sphericity was violated, χ^2^(5) = 9.503, *p* = 0.091; therefore, degrees of freedom were corrected using Greenhouse–Geisser estimates of sphericity (ε = 0.845). The results showed a significant main effect of window size on perceived restorativeness in virtual reality settings, F(2.536, 86.223) = 84.286, *p* < 0.001, partial η^2^ = 0.713. Post hoc tests using the Bonferroni correction revealed that perceived restorativeness significantly increased as the window size increased, with all pairwise comparisons being statistically significant (*p* < 0.001) except between the 33% and 66% window conditions (*p* = 0.222). These findings support the hypothesis that spaces with larger windows would be evaluated as significantly more restorative compared with spaces with smaller windows or no openings.

#### 7.2.2. View Content

H3_Screen: Particular natural features (e.g., water elements, diverse vegetation) will demonstrate a stronger positive correlation with perceived sense of restoration compared with other natural elements.

Mauchly’s test indicated that the assumption of sphericity had been violated, χ^2^(5) = 15.692, *p* = 0.008; therefore, degrees of freedom were corrected using Greenhouse–Geisser estimates of sphericity (ε = 0.746). The results showed a significant main effect of view content on perceived restorativeness, F(2.237, 76.041) = 11.848, *p* < 0.001, partial η^2^ = 0.258. Post hoc tests using the Bonferroni correction revealed that water views were perceived as significantly more restorative than desert (*p* < 0.001) or lawn views (*p* < 0.001). Wooded views were also rated significantly more restorative than desert (*p* = 0.023) or lawn views (*p* < 0.001). There were no significant differences between water and wooded views (*p* = n.s.) or between desert and lawn views (*p* = n.s.). These findings partially support the hypothesis that particular natural features, specifically water and diverse vegetation, are associated with a stronger perceived sense of restoration compared with other natural elements.

H3_VR: Particular natural features (e.g., water elements, diverse vegetation) will demonstrate a stronger positive correlation with perceived sense of restoration compared with other natural elements.

Mauchly’s test indicated that the assumption of sphericity was not violated, χ^2^(5) = 9.342, *p* = 0.096. The results showed a significant main effect of view content on perceived restorativeness, F(3, 102) = 11.640, *p* < 0.001, partial η^2^ = 0.255. Post hoc tests using the Bonferroni correction revealed that water views in VR were perceived as significantly more restorative than desert (*p* = 0.001), wooded (*p* = 0.019), or lawn views (*p* < 0.001). There were no significant differences between desert and wooded views (*p* = n.s.), desert and lawn views (*p* = 0.396), or wooded and lawn views (*p* = 0.128). These findings partially support the hypothesis that particular natural features in VR, specifically water elements, demonstrate a stronger perceived sense of restoration compared with other natural elements.

#### 7.2.3. Materiality

H4_Screen: Interior environments incorporating biophilic features and sensory-rich materials (including natural materials, textures, and patterns) will generate significantly higher restoration ratings compared with alternative material schemes.

Mauchly’s test indicated that the assumption of sphericity was not violated, χ^2^(5) = 7.752, *p* = 0.171. The results showed a significant main effect of materiality on perceived restorativeness, F(3, 102) = 21.154, *p* < 0.001, partial η^2^ = 0.384. Post hoc tests using the Bonferroni correction revealed that natural materials were perceived as significantly more restorative than concrete (*p* < 0.001), resilient finishes (*p* < 0.001), or brick (*p* = 0.854, n.s.). Brick was rated significantly more restorative than concrete (*p* < 0.001) and resilient materials (*p* = 0.002). There was no significant difference between concrete and resilient materials (*p* = n.s.). These findings support the hypothesis that settings with materials providing sensory richness and biophilic design features, particularly natural materials, would be evaluated as significantly more restorative compared with other settings.

H4_VR: Interior environments incorporating biophilic features and sensory-rich materials (including natural materials, textures, and patterns) will generate significantly higher restoration ratings compared with alternative material schemes.

Mauchly’s test indicated that the assumption of sphericity was not violated, χ^2^(5) = 10.847, *p* = 0.055. The results showed a significant main effect of materiality (i.e., room finish) on perceived restorativeness, F(3, 102) = 23.120, *p* < 0.001, partial η^2^ = 0.405. Post hoc tests using the Bonferroni correction revealed that natural materials were perceived as significantly more restorative than concrete (*p* < 0.001) or resilient finishes (*p* < 0.001), but not brick materials (*p* = n.s.). Brick materials were rated significantly more restorative than concrete (*p* < 0.001) or resilient materials (*p* = 0.002). The difference between concrete and resilient materials approached significance (*p* = 0.067). These findings support the hypothesis that settings with materials providing sensory richness and biophilic design features, particularly natural materials, would be evaluated as significantly more restorative compared with other settings when experienced in VR.

#### 7.2.4. Room Geometry

H5_Screen: Non-flat ceiling designs (vaulted, curved, or angled configurations) will generate significantly higher perceived restoration levels than flat ceiling conditions.

Mauchly’s test indicated that the assumption of sphericity was not violated, χ^2^(5) = 2.907, *p* = 0.714. The results showed a significant main effect of room geometry on perceived restorativeness, F(3, 102) = 12.949, *p* < 0.001, partial η^2^ = 0.276. Post hoc tests using the Bonferroni correction revealed that the vaulted, angled, and curved ceiling designs were perceived as significantly more restorative than the flat ceiling (*p* < 0.001, *p* < 0.001, and *p* = 0.002, respectively). There were no significant differences between the vaulted and angled ceilings (*p* = 0.226), vaulted and curved ceilings (*p* = 0.196), or angled and curved ceilings (*p* = 1.000). These results support the hypothesis that non-flat ceiling designs (vaulted, curved, or angled configurations) would generate significantly higher perceived restoration ratings compared with flat ceiling conditions.

H5_VR: Non-flat ceiling designs (vaulted, curved, or angled configurations) will generate significantly higher perceived restoration levels than flat ceiling conditions.

Mauchly’s test indicated that the assumption of sphericity was not violated, χ^2^(5) = 8.926, *p* = 0.112. The results showed a significant main effect of VR room geometry on perceived restorativeness, F(3, 102) = 9.254, *p* < 0.001, partial η^2^ = 0.214. Post hoc tests using the Bonferroni correction revealed that the vaulted ceiling design was perceived as significantly more restorative than the flat ceiling (*p* < 0.001), angled ceiling (*p* = 0.046), or curved ceiling (*p* = 0.002). There were no significant differences between the flat and curved ceilings (*p* = 0.467), flat and angled ceilings (*p* = 0.841), or angled and curved ceilings (*p* = n.s.). These findings partially support the hypothesis that when experienced in VR, rooms with varied ceiling designs would be perceived as significantly more restorative compared with the room with a flat ceiling, with the vaulted ceiling design showing the strongest effect.

## 8. Discussion

This study investigated the effects of various environmental design elements on perceived restorativeness in both screen-based and virtual reality (VR) presentations. The research examined four key variables: view access, view content, materiality, and room geometry. The findings provide valuable insights into how these elements influence the restorative potential of interior environments.

### 8.1. Comparison of Screen-Based and VR Presentations

The first hypothesis (H1) proposed that perceived restorativeness would be significantly higher in VR presentations compared with screen-based ones. This hypothesis was partially supported, with VR eliciting higher restorativeness scores for some environments, particularly those with natural elements or rich textures. However, the effect was not consistent across all stimuli, suggesting that the relationship between presentation mode and perceived restoration was context-dependent.

These mixed results align with previous research, highlighting the complex relationship between virtual environments and perceived restoration. The findings suggest that while VR can enhance the perception of restorative qualities in some cases, it may not universally outperform traditional screen-based presentations. This underscores the importance of considering the specific environmental context when designing virtual restorative environments.

### 8.2. View Access

The second hypothesis (H2) posited that spaces with larger windows would be evaluated as significantly more restorative than those with smaller windows or no openings. This hypothesis was strongly supported in both the screen-based and VR presentations, with a clear linear trend of increasing restorativeness as window size increased.

These findings corroborate existing literature on the positive effects of view access on well-being and restoration [24]. The results underscore the importance of incorporating ample window space in interior design, particularly in settings where restoration is a priority, such as healthcare facilities.

### 8.3. View Content

The third hypothesis (H3) suggested that particular natural features, such as water elements and diverse vegetation, would demonstrate a stronger perceived sense of restoration compared with other natural elements. This hypothesis was partially supported. The view of water was consistently rated as the most restorative across both presentation modes, but the relationships between other natural elements varied between the screen-based and VR presentations. These results align with previous research by White et al. (2010), which found that aquatic environments were rated more positively for restoration than green spaces alone [55]. However, the current findings also reveal nuances in the restorative potential of different natural elements, with wooded areas showing higher restorativeness than desert or lawn views in screen-based presentations, but not in VR. These differences might have beenb due to the differences in cultural backgrounds and experiences of the participants.

### 8.4. Materiality

The study’s fourth hypothesis (H4) suggested that interior settings characterized by biophilic features and sensory-rich materials would yield significantly higher perceived restorativeness compared with alternative material schemes.

This hypothesis was supported by both the screen-based and VR presentations, with natural materials consistently rated as the most restorative. These findings reinforce the principles of biophilic design [31] and highlight the importance of materials selection in creating restorative environments.

### 8.5. Room Geometry

The fifth hypothesis (H5) suggested that rooms with non-flat ceiling designs would be perceived to be significantly more restorative compared with flat ceilings. This hypothesis was supported by the screen-based presentations and partially supported by the VR presentations.

In the screen-based presentations, all varied ceiling designs (vaulted, curved, and angled) were perceived as more restorative than the flat ceiling. However, in VR, only the vaulted ceiling design showed a significantly higher restorativeness rating. Previous studies have suggested that curvilinear architectural forms are preferred. These findings contribute to the growing body of research on the impact of spatial geometry on environmental preference and restoration [61,62].

## 9. Limitations

Several limitations of this study should be noted. First, while the use of a single-item measure for perceived restorativeness offered practical advantages in terms of reducing participant fatigue, especially in terms of minimizing potential cybersickness during repeated VR exposure, it involved certain methodological constraints. Although single-item measures have demonstrated validity in architectural and design research, multi-item scales such as the shortened perceived restorativeness scale [71] can provide further insights into different aspects of restoration. The single-item approach in this study may have made the study’s focus more apparent to the participants.

The sequential presentation of stimuli, while necessary for maintaining logical coherence in the progression of environmental changes (particularly for view access and materiality), could have introduced order effects. While randomization might have reduced these effects, it would have complicated the analysis of relationships between incrementally changing environmental features and could have disrupted participants’ ability to make meaningful comparative judgments.

A methodological consideration emerged in the manipulation of the room geometry. While consistent window dimensions were maintained across all ceiling configurations, the ceiling variations resulted in slight differences in the total wall area housing the window. The flat ceiling configuration provided 74.7% wall area around the window, the vaulted ceiling 71.3%, the curved ceiling 71.6%, and the angled ceiling 69.2%. Given these relatively minor variations in the wall area and the consistent window size, the observed differences in restorativeness ratings can be reasonably attributed to the manipulation of ceiling geometry.

The study examined each of the four variables (view access, view content, materiality, room geometry) independently rather than in a fully crossed design, due to the number of conditions and levels in each set.

A methodological limitation arose from the different interaction capabilities between the presentation modes. While VR participants could naturally rotate their heads to explore the environment, screen-based presentations had fixed viewpoints to enable precise eye-tracking measurements focusing on attention patterns and visual behavior. The findings from the eye-tracking analyses will be reported in a separate manuscript.

Additionally, the sample’s gender distribution (92% female) was notably uneven, which may limit generalizability. While participants included both undergraduate and graduate students, future research should examine these effects across more gender-balanced populations to ensure broader applicability of the findings.

Finally, this study’s focus on immediate responses to environmental stimuli did not capture longer-term restorative effects. Future research could benefit from longitudinal designs that examine how different presentation modes affect restoration over extended periods.

## 10. Conclusions

The results of this study have significant implications for the design of restorative environments in both physical and virtual settings. The findings underscore the importance of integrating natural elements and maximizing access to views in interior spaces. Future research could further explore the relationship between opening size and sense of safety and privacy, as larger openings can potentially influence users’ sense of enclosure, indirectly affecting their sense of restoration. This is particularly crucial in centers for mental health treatment, where the balance between openness and privacy is essential for patient well-being.

Materiality and view content play important roles in place attachment and the attribution of contextual valuations to particular spaces. For instance, medical treatment centers are often perceived as comparatively sterile environments. However, the integration of natural materials and restorative views could potentially alter these perceptions, creating more comforting and healing spaces. In the context of mental health treatment in VR, HDR views of particular environments, as suggested by medical providers, could potentially help in alleviating triggering stimuli or gradually exposing patients to anxiety-inducing scenes in a controlled manner.

While the settings in this study were abstract and hypothetical, with controlled variables, it is important to note that the findings might have been affected by participants’ intrinsic aesthetic judgments or biases. As previous research suggests, extracting emotional experiences from study stimuli necessitates sufficient time for participants to engage with the environment. However, longer data collection sessions may lead to carry-over effects, potentially influencing subsequent evaluations. This presents a methodological challenge that future studies should address.

To mitigate these carryover effects and gain more nuanced insights, future research could incorporate advanced VR technologies and assessment methods. For instance, the use of VR headsets equipped with eye-tracking capabilities could provide valuable data on participants’ visual attention patterns and cognitive load during exposure to different restorative environments. Eye-tracking metrics such as fixation duration, saccade patterns, and pupil dilation could offer objective measures of cognitive processing and emotional responses, complementing self-reported restorativeness scores [72].

Cognitive load assessment using VR eye tracking could help researchers understand how different design elements impact mental effort and information processing. This could be particularly relevant when studying the restorative potential of environments for individuals with varying cognitive capacities or those undergoing mental health treatment. By analyzing gaze patterns and cognitive load indicators, researchers could identify which environmental features are most effective in promoting restoration while minimizing mental strain for patients.

The VR findings in the current study provide valuable insights, but they also raise questions for future research. For instance, the heightened restorativeness scores in VR for some variables suggest that virtual environments might offer enhanced restorative experiences. However, it is crucial to investigate whether these effects persist over extended periods and are replicable when users perceive the space from different viewpoints.

Additionally, this study’s findings on room geometry, particularly the positive impact of ceiling design in VR, open up new opportunities for architectural design in both virtual and physical spaces. Future research could investigate how different spatial configurations, volume, and scale affect restoration, potentially leading to innovative design solutions in healthcare, workplace, and recreational environments.

## Figures and Tables

**Table 1:** Perceived restorativeness ratings and correlations between screen-based and VR ratings (N = 35).

	Screen	VR	
	Perceived Restorativeness–*M (SD)*	Perceived Restorativeness–*M (SD)*	Pearson Correlations–ρ
**Set 1: View Access**			
0%	3.89 (1.301)	3.26 (1.482)	0.443 **
33%	4.80 (0.994)	5.11 (1.105)	0.262
66%	5.40 (0.812)	5.51 (0.951)	0.526 **
100%	6.37 (0.690)	6.51 (0.818)	0.538 **
**Set 2: View Content**			
Desert	4.23 (1.457)	4.77 (1.352)	0.654 **
Water	5.11 (1.491)	5.54 (1.268)	0.433 **
Woods	4.83 (0.985)	4.80 (1.208)	0.613 **
Lawn	4.06 (0.998)	4.34 (1.235)	0.556 **
**Set 3: Materiality**			
Brick	4.06 (1.282)	4.60 (1.376)	0.680 **
Concrete	2.77 (1.262)	2.86 (1.498)	0.791 **
Natural	4.46 (1.245)	4.80 (1.346)	0.811 **
Resilient	2.94 (1.056)	3.40 (1.355)	0.407 *
**Set 4: Room Geometry**			
Curve	3.89 (1.278)	3.94 (1.187)	0.713 **
Flat	3.23 (1.330)	3.63 (1.330)	0.648 **
Angled	3.94 (1.211)	3.94 (1.282)	0.471 **
Vault	4.29 (1.202)	4.60 (1.218)	0.663 **

Note: * = *p* < 0.05 (2-tailed). ** = *p* < 0.01 level (2-tailed).

**Table 2:** Repeated-Measures ANOVA Results for the Effect of View Access, View Content, Materiality, and Room Geometry on Perceived Restorativeness, Screen-Based Evaluations.

Source	*SS*	*df*	*MS*	*F*	*p*	*η* ^2*p*^	*ε*
View Access	114.457	2.095	54.630	59.374	<0.001	0.636	0.698
Error	65.543	71.234	0.920				
View Content	25.971	2.237	11.613	11.848	<0.001	0.258	0.746
Error	74.529	76.041	0.980				
Materiality	71.914	3	23.971	21.154	<0.001	0.384	
Error	115.586	102	1.133				
Room Geometry	20.479	3	1.740	12.949	<0.001	0.276	
Error	53.771	102	0.662				

Note: *SS* = sum of squares; *df* = degrees of freedom; *MS* = mean square; *η*^2*p*^ = partial eta squared; *ε* = Greenhouse–Geisser correction, if needed.

**Table 3:** Repeated-Measures ANOVA Results for the Effect of View Access, View Content, Materiality, and Room Geometry on Perceived Restorativeness, VR Evaluations.

Source	*SS*	*df*	*MS*	*F*	*p*	*η* ^2*p*^
View Access	194.886	3	64.962	84.286	<0.001	0.713
Error	78.614	102	0.771			
View Content	26.079	3	8.693	11.640	<0.001	0.255
Error	76.171	102	0.747			
Materiality	92.286	3	30.762	23.120	<0.001	0.405
Error	135.714	102	1.631			
Room Geometry	17.543	3	5.848	9.254	<0.001	0.214
Error	64.457	102	0.632			

Note: *SS* = sum of squares; *df* = degrees of freedom; *MS* = mean square; *η*^2*p*^ = partial eta squared.

**Table 4:** Results from pairwise comparisons with Bonferroni correction.

Variables	Mean Difference	*SE*	*p*
***Screen-based evaluations***			
**Set 1: View Access**			
P1: 0–33%	−0.914 *	0.237	0.003
P2: 0–66%	−1.514 *	0.240	<0.001
P3: 0–100%	−2.486 *	0.202	<0.001
P4: 33–66%	−0.600 *	0.131	<0.001
P5: 33–100%	−1.571 *	0.165	<0.001
P6: 66–100%	−0.971 *	0.145	<0.001
**Set 2: View Content**			
P1: Desert–Water	−0.886 *	0.168	<0.001
P2: Desert–Woods	−0.600 *	0.193	0.023
P3: Desert–Lawn	0.171	0.226	1.000
P4: Water–Woods	0.286	0.223	1.000
P5: Water–Lawn	1.057 *	0.246	<0.001
P6: Woods–Lawn	0.771 *	0.154	<0.001
**Set 3: Materiality**			
P1: Brick–Concrete	1.286 *	0.248	<0.001
P2: Brick–Natural	−0.400	0.266	0.854
P3: Brick–Resilient	1.114 *	0.283	0.002
P4: Concrete–Natural	−1.686 *	0.277	<0.001
P5: Concrete–Resilient	−0.171	0.211	1.000
P6: Natural–Resilient	1.514 *	0.233	<0.001
**Set 4: Room Geometry**			
P1: Curve–Flat	0.657 *	0.164	0.002
P2: Curve–Angled	−0.057	0.188	1.000
P3: Curve–Vault	−0.400	0.180	0.196
P4: Flat–Angled	−0.714 *	0.167	<0.001
P5: Flat–Vault	−1.057 *	0.183	<0.001
P6: Angled–Vault	−0.343	0.158	0.226
***VR evaluations***			
**Set 1: View Access**			
P1: 0–33%	−1.857 *	0.232	<0.001
P2: 0–66%	−2.257 *	0.237	<0.001
P3: 0–100%	−3.257 *	0.240	<0.001
P4: 33–66%	−0.400	0.184	0.222
P5: 33–100%	−1.400 *	0.197	<0.001
P6: 66–100%	−1.000 *	0.153	<0.001
**Set 2: View Content**			
P1: Desert–Water	−0.771 *	0.184	0.001
P2: Desert–Woods	−0.029	0.223	1.000
P3: Desert–Lawn	0.429	0.226	0.396
P4: Water–Woods	0.743 *	0.233	0.019
P5: Water–Lawn	1.200 *	0.178	<0.001
P6: Woods–Lawn	0.457	0.189	0.128
**Set 3: Materiality**			
P1: Brick–Concrete	1.743 *	0.294	<0.001
P2: Brick–Natural	−0.200	0.249	1.000
P3: Brick–Resilient	1.200 *	0.306	0.002
P4: Concrete–Natural	−1.943 *	0.290	<0.001
P5: Concrete–Resilient	−0.543	0.202	0.067
P6: Natural–Resilient	1.400 *	0.299	<0.001
**Set 4: Room Geometry**			
P1: Curve–Flat	0.314	0.173	0.467
P2: Curve–Angled	0.000	0.169	1.000
P3: Curve–Vault	−0.657 *	0.164	0.002
P4: Flat–Angled	−0.314	0.208	0.841
P5: Flat–Vault	−0.971 *	0.186	<0.001
P6: Angled–Vault	−0.657 *	0.232	0.046

Note: * mean difference significant at the 0.05 level.

## Data Availability

Data will be available to interested researchers upon request.
